# The cost of care for people living with stable HIV in Croatia and the efficiency of EmERGE

**DOI:** 10.3325/cmj.2021.62.542

**Published:** 2021-12

**Authors:** Eduard Beck, Sundhiya Mandalia, Platonas Yfantopoulos, Christopher Jones, Stephen Bremner, Jennifer Whetham, Ivana Benković, Šime Zekan, Josip Begovac

**Affiliations:** 1 NPMS-HHC Community Interest Company, London, United Kingdom; 2 Department of Health Services Research and Policy, Faculty of Public Health & Policy, London School of Hygiene & Tropical Medicine, London, United Kingdom; 3 Department of Primary Care and Public Health, Brighton and Sussex Medical School, Brighton, United Kingdom; 4 Lawson Unit, Brighton and Sussex University Hospitals Trust and Medical School, Brighton, United Kingdom; 5 HIV Department, University Hospital for Infectious Diseases, Zagreb, Croatia; 6 Reference Center for Diagnostics and Treatment of HIV-infection, Dr Fran Mihaljević University Hospital for Infectious Diseases, Zagreb, Croatia

## Abstract

**Aim:**

To estimate the cost-effectiveness of the EmERGE Pathway of Care for medically stable people living with HIV (PLHIV) at the University Hospital for Infectious Diseases (UHID), Zagreb. The Pathway includes a mobile application enabling individuals to communicate with their caregivers.

**Methods:**

This study involving 293 participants collected data on the use of HIV outpatient services one year before and after EmERGE implementation. In departments supporting HIV outpatients, a micro-costing exercise was performed to calculate unit costs. These were combined with mean use of HIV services per patient year (MPPY) to estimate average annual costs. Primary outcomes were CD4 count, viral load, and secondary outcomes were patient activation, PAM13; and quality of life, PROQOL-HIV. Information on out-of-pocket expenditures was also collected.

**Results:**

Outpatient visits decreased by 17%, from 4.0 (95% CI 3.8-4.3) to 3.3 MPPY (95% CI 3.1-3.5). Tests, including CD4 count, decreased, all contributing to a 33% reduction of annual costs: 7139 HRK (95% CI 6766-7528) to 4781 HRK (95% CI 4504-5072). Annual costs including anti-retroviral drugs (ARVs) decreased by 5%: 43 101 HRK (95% CI 42 728-43,490) to 40 743 HRK (95% CI 40 466-41,034). ARVs remain the main cost driver in stable PLHIV. Primary and secondary outcomes did not change substantially between periods.

**Conclusion:**

EmERGE Pathway was a cost-saving intervention associated with changes in management, and a reduction in outpatient visits, tests, and costs. ARV costs dominated costs. Future efficiencies are possible if EmERGE is introduced to other PLHIV across the UHID and if ARV prices are reduced.

Life-expectancy of people living with HIV (PLHIV) now approximates that of those not living with HIV (1), largely due to the global roll-out of antiretroviral drugs (ARV) (2). This will increase the number of PLHIV, including those aged 50 years or older (3). The commonest causes of death for PLHIV in high income countries are non-HIV cancers, cardiovascular disease, and other non-communicable diseases (NCDs), and these diseases have become increasingly prevalent among PLHIV in low- and middle-income countries (4,5). PLHIV will therefore increasingly need to use both HIV and non-HIV health and social services.

Emphasis has been placed on tracking the use of health services by PLHIV, across health care sectors and monitor and evaluate their use, cost, outcome, and impact over time in countries at individual and health system levels (6). Improved health information systems can integrate health-services and make them more cost-effective (7). Mobile Health (mHealth) now plays an increasingly important role in linking and integrating health services, using wireless technology to deliver information and health services via mobile communication devices including mobile phones, tablet computers, smartphones, and other devices (8,9). The coronavirus 2019 (COVID-19) pandemic has increased the use of all forms of telemedicine, including mHealth (10), which is now used to manage people living with chronic illnesses including cancer. These strategies are likely to continue beyond the pandemic (11).

The *Evaluating mHealth technology in HIV to improve Empowerment and health care utilization: Research and innovation to Generate Evidence for personalised care* (EmERGE) Project (12) developed a new digital mHealth pathway, which was implemented in five HIV clinics in five European countries: Belgium, England, Portugal, Spain, and Croatia. The mobile health application (App) provides personal health information to its users, people living with medically stable HIV, and enables communication with caregivers.

A recent review identified nine communication functions required by an mHealth App (9), but this did not include two important aspects (Table 1): data collected, transmitted, and stored on phones or server(s) need to have their confidentiality and security protected, and such technology needs to be affordable and efficient (13).

**Table 1:** Eleven requirements of a tele-medicine system

Requirements
1. Patient-provider and peer communication*
2. Medication and appointment reminders*
3. A medication checklist, pill identification function, and list of current and discontinued medicines*
4. Laboratory reports (CD4 count, viral load, sexually transmitted infections, glucose and complete blood count)*
5. Pharmacy information*
6. Nutrition and fitness trackers*
7. Resources, links to social services, substance abuse support, video testimonials, case-management*
8. Settings (profile picture, password and alerts)*
9. A search function*
10. Protecting the confidentiality and security of personal information at rest and in-transit^†^
11. Affordability and efficiency of the technology^†^

*(9).

†(13).

Most mHealth studies to date have not included the cost for developing and implementing mHealth Pathways, either their cost-effectiveness or cost-savings. The need for such efficiency studies was recognized a long time ago (14,15), but few efficiency studies have been performed since then (16).

The aim of this study was to calculate the efficiency of the implementation of the EmERGE pathway at the University Hospital for Infectious Diseases (UHID), the National Reference Centre for HIV/AIDS in Zagreb, Croatia. The specific objectives were: 1) to calculate the use of services by participants one year before and after EmERGE implementation; 2) to calculate the unit costs of HIV outpatient services for EmERGE participants; 3) to calculate the annual costs of these outpatient services for participants one year before and after EmERGE implementation; 4) to calculate the efficiency of the implementation of the EmERGE Pathway at UHID.

## Methods

Initially, 309 study participants were recruited based on specified selection criteria (Table 2), and 293 were followed up between May 1, 2016 and October 30, 2018. The “before-and-after” method was used to estimate the efficiency of the intervention (18).

**Table 2:** Inclusion and exclusion criteria for EmERGE Participants

Documented HIV infection
**Inclusion criteria**
Aged at least 18 years old
Able to give informed consent
In possession of a smartphone, tablet, or similar technology supporting the mHealth platform
Clinically stable on anti-retroviral therapy (ART). This was defined as receiving ART for at least 1 year and unchanged regimen for at least 3 months, 2 consecutive undetectable viral load measures (<50 copies/mL), no current pregnancy and without any new WHO clinical stage 2, 3, or 4 events within the previous 12 months*.
Exclusion criteria
Aged less than 18 years
Pregnant
Participating in a clinical trial or receiving an investigational medication
Unable to comprehend the patient information sheet
Unable to comprehend the instructions for using the mHealth platform
Considered for any other reason by their regular physician to be unsuitable for study participation

*(17).

### Context

EmERGE study participants were all managed in the HIV outpatient clinic: only 12 (4%) of 293 participants had an inpatient episode, none used the day-care hospital, and none underwent a surgical procedure. The HIV outpatient clinic was therefore the focus of the micro-costing study. The clinic employs doctors and nurses, psychologists, and social workers.

ARV prescriptions were provided for 3-6 months depending on the type of drugs, the patients' adherence, and the distance they lived from the clinic. ARV medication was free at point of delivery. When laboratory tests were ordered, bloods were drawn and transported to the laboratories. Results were sent back to the consultant and copied to the Billing Department of the UHID for re-imbursement from the Croatian Health Insurance Fund.

Most patients had their tests performed on the same day when seeing their consultant. Results were discussed on a follow-up face-to-face visit or virtual visit with their physician. Some patients combined their consultant visit with collecting their ARVs at the hospital, while others only collected ARVs. ARVs could also be delivered, but few patients used this option as they were charged a delivery fee.

As per EmERGE protocol, one visit per year was an electronic “visit” when the physician reviewed the results of blood tests electronically and sent their recommendation for future treatment via the EmERGE App; the other annual visit, part of routine management, was a face-to-face visit.

### Data collected

Data on the annual use, cost, and outcome of HIV services were collected before and after EmERGE implementation. Process information on the use of services by individual EmERGE participants was combined with unit cost data of the services used, and changes in primary or secondary outcome measures.

*Costing health facilities.* All departments that provided services for EmERGE participants were identified and costed. The main methods for micro-costing studies are the “top-down” and “bottom-up or ingredient-based” approaches (19,20). “Bottom-up/ingredients-based” studies define the type and quantity of inputs used to produce the service output. The costs of all inputs are added and divided by the number of “products” produced to obtain the unit cost for each product or “output” of that service. The “product” includes all services provided, including an inpatient day, outpatient visit, tests performed, etc (19,20). The “top-down” method is easier to perform and determines the total expenditure of providing services on account of past expenditure. This expenditure is then divided by the number of “products” provided during the period. While the ingredients-based method is preferred, its execution depends on the availability of detailed data. In this study, where possible, this approach was applied.

*Process data.* The individual data on the use of services by a participant in the year before and after EmERGE implementation comprised the process data that identified which services were used and should be costed. As EmERGE participants were medically stable, they predominantly used outpatient services, the focus of the micro-costing exercise.

*Unit costs data.* The costing study was based on the UNAIDS Costing Manual (19) and UNAIDS Costing Workbook (20). Departmental workload and financial data were collected from the departments supporting outpatient services for EmERGE participants. Departmental process data were the workload generated by EmERGE participants. Departmental financial data were collected using the SCOPE framework and included the following categories: Staff, Consumables, Overhead, Procedures, and Equipment costs (21). The estimated unit costs of outpatient services were combined with individual-level process data to calculate the annual costs of outpatient services (22).

The departments supporting EmERGE outpatient services were the Biochemistry, Hematology and Immunology and Molecular Diagnostics laboratories, the Radiology Department, and Pharmacy. Two overhead departments were costed: Department of Buildings and Maintenance, or Technical Department, and the Management and Administration Department.

The staff of the Department of Buildings and Maintenance performed maintenance and repairs of the hospital buildings. As they serviced many buildings within the Hospital, the weight used to apportion this service was the area in square meters (m^2^) of each of the departments serviced by this Department, divided by the area in square meters of the whole facility (21).

The Management and Administration Department was responsible for the general management of the Hospital, including legal support for Hospital employees, human resources teams responsible for employment issues, finance and accounting functions responsible for making payments and handling financial accounts, processing bills for the National Insurance reimbursements, storage of office items used around the hospital, and medical records. All these supporting functions involved human resources. For this reason, the costs of running this Department were apportioned by the full-time equivalent (FTE) staff of each department divided by the FTE of the whole Hospital (21).

Cleaning for the whole Hospital was performed by cleaners, and each department had its own team of cleaners. Departments paid for the purchase of materials needed for cleaning. Cleaning costs were added to every individual department, weighted by the ratio of the respective departmental areas in square meters divided by the total square meters of the Hospital (21).

### Statistical analysis

Summary statistics are presented with point estimates and indication of variability and missing data. Linear mixed models were used to calculate difference in averages (DAVG). Time-weighted changes of CD4 count and viral load were analyzed over a two-year period and measured by time point changes during one year before recruitment to EmERGE, and one year after recruitment (23). MIXED procedure in SAS was used by fitting routine values of CD4 counts and viral loads results as dependent variables. Independent variables included the fixed effects of study visit time points. A covariance matrix was used to model the within-patient errors. Estimates of effects are based on MIXED models and assume any missing data were missing at random. Trends over time are presented as point estimates derived from the models. Viral load data were transformed logarithmically to stabilize their variance.

The mean number of services used per patient year (MPPY) was calculated using methods employed previously (24-26), based on the following formula:


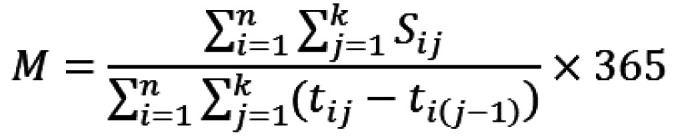


Where n = total number of individuals; k = day of censoring; S_ij_ = use of service by individual i on j^th^ day; t_ij_ = number of days of follow-up for individual i; M = mean of services S per patient-year.

The denominator comprised the total duration of follow-up for all patients during a calendar year, from when they entered the study to one year after study entry. The data were censored at the one-year pre-baseline visit. Post-mHealth data were right-censored at either one year since baseline visit if patients were still under follow-up at one year, or at their date of death if they had died during follow-up, or if they were lost to follow-up, whichever came first. Numerators were calculated by summing the use of outpatient services and mean use of services PPY (Equation 1.1). Exact Poisson 95% confidence intervals (CIs) were estimated for MPPY based on the distribution of the observed number of outpatient visits divided by the total duration of follow-up for all patients during a calendar year. All statistical analyses were performed using SAS version 9.4 (27).

Average annual costs per patient-year (PPY) of HIV outpatient services for EmERGE participants were produced by multiplying MPPY outpatient visits by their respective unit costs. The total annual costs for providing services were obtained by adding the annual costs for outpatient visits, with the annual cost of tests, drugs, and procedures performed at the UHID for EmERGE participants (21). Costings were performed from a societal perspective.

### Primary and secondary outcome measures

The primary outcome measures were CD4 count and viral loads before and after EmERGE. Secondary outcome measures included changes to PAM-13, index of patient activation (28), and PROQOL-HIV, quality of life measure (29), observed between month 0 (baseline) and month 12 after EmERGE implementation.

### Cost-effectiveness analyses

Incremental cost-effectiveness ratios (ICERs) were used to calculate the cost-effectiveness or cost-saving based on changes in annual costs, and primary and secondary outcome measures before and after EmERGE implementation (30):


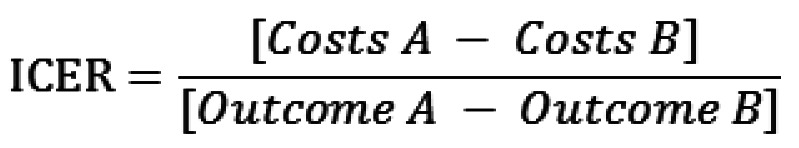


Both primary and secondary outcome measures did not change substantially, and the study ended up being a cost-minimization study comparing annual costs between periods.

### Out of pocket expenditure

Information was also collected on the socio-economic status of EmERGE participants, time-off-work for clinic appointments, return traveling time, and costs for clinic appointments.

## Results

Of the 309 recruited participants, 293 were followed up prospectively between May 1, 2016 and October 30, 2018. Sixteen participants were excluded. One woman became pregnant during the study period, two deaths occurred, three participants relocated and were managed at a different clinical site, three dropped out, and seven people did not use the App. Some of the 16 excluded participants did complete some of the first PAM-13 and PROQOL-HIV questionnaires and these were retained for analyses.

Of the remaining 293 participants, 90% were men. All participants self-identified as white. Mean age at study entry was 41.4 years (95%CI 39.9-41.9). Eighty percent were men who have sex with men, 8% heterosexual, 6% bisexual, and 7% were other. Three persons had injected themselves with drugs in the past.

At recruitment, 79% of participants were fully employed, with a median 40-hour workweek (IQR 40-45 hours) and a median monthly income of 6000 HRK (IQR 4500- 8000 HRK). The remaining 21% were either retired, unemployed, or receiving social benefits: 11% of participants received income support, 10% housing benefits, and 5% pension credits. The median monthly income for them was 1059 HRK (IQR 500-1560 HRK). The median number of sick days was 0 days (IQR 0-0) three months before enrolment, while 5 participants were on sick-leave and two were disabled. To visit the clinic, 41% of participants took a day-off, and the median return travel time to the clinic was 1.5 hours (IQR 0.5-2.5 hours); the median cost of this return journey was 138 HRK (IQR 20-300 HRK).

### Estimated unit costs

The unit cost data estimated through the micro-costing exercise (21) are displayed in Table 3.

**Table 3:** Estimated unit costs for University Hospital for Infectious Diseases (UHID) HIV outpatient services, 2017 financial data (21)

UHID	Cost/Unit cost (HRK)
Unit cost per EmERGE Outpatient clinic visit	363
Pharmacy Department Costs:	
annual Pharmacy costs excluding drugs per EmERGE participant	418
annual Pharmacy cost of ARVs per EmERGE participant	35 544
total Annual Pharmacy costs per EmERGE participant	35 962
Unit cost per EmERGE patient	
Biochemistry Laboratory test	9
Hematology Laboratory test	46
Viral Load test (PCR)	543
CD4 test (Cytometry)	522
Radiology investigation	3

### Annual use and cost of services pre- and post-EmERGE

Outpatient service was the main service used, and the number of annual visits decreased by 17%, from 4.0 MPPY (95%CI 3.8-4.3) to 3.3 MPPY (95%CI 3.1-3.5). The MPPY of tests and radiology examinations performed after implementation also decreased (Table 4).

**Table 4:** Annual use and costs of outpatient services pre- and post-EmERGE at University Hospital for Infectious Diseases

	Pre-EmERGE			Post EmERGE		
		Lower CI	Upper CI		Lower CI	Upper CI
Mean outpatient visits PPY	4.0	3.8	4.3	3.3	3.1	3.5
Average annual costs outpatient visits (HRK)	1456	1369	1550	1198	1122	1278
Mean biochemistry tests PPY	49.5	48.6	50.4	41.2	40.5	42.0
Average annual costs biochemistry tests (HRK)	446	438	454	371	364	378
Mean hematology tests PPY	32.2	31.5	32.9	26.5	25.9	27.1
Average annual hematology tests costs (HRK)	1479	1447	1512	1219	1191	1247
Mean viral load tests PPY	2.9	2.7	3.1	2.4	2.2	2.6
Average annual viral load tests costs (HRK)	1581	1466	1700	1314	1216	1417
Mean CD4 count tests PPY	4.2	3.9	4.4	1.3	1.2	1.4
Average annual CD4 cell count tests costs (HRK)	2177	2046	2312	679	611	752
Mean Radiology tests PPY	0.06	0.04	0.10	0.04	0.02	0.07
Average annual radiology tests costs (HRK)	0.18	0.12	0.30	0.12	0.06	0.21
Total annual cost outpatient services (HRK)	7139	6766	7528	4781	4504	5072
Average annual ARV costs (HRK)	35 544					
Average additional pharmacy costs (HRK)	418					
Average total annual cost including ARVs (HRK)	43 101	42 728	43 490	40 743	40 466	41 034

*Abbreviations: CI – confidence interval; PPY – per patient year; ARV – anti-retroviral drugs.

The reduction in outpatient visits and tests resulted in a 33% reduction in average annual outpatient costs, from 7139 HRK PPY (95%CI 6675-7528 HRK) to 4781 HRK PPY (95%CI 4504 -5072 HRK; Table 4). The average annual cost for ARVs was 35 544 HRK, and overall pharmacy cost was 35 962 HRK PPY (Table 4). The overall average annual costs including ARV decreased by 5%, from 43 101 HRK PPY (95%CI 42 728-43,490) to 40 743 HRK PPY (95%CI 40 466-41,034).

Tests and associated costs also decreased. In particular, the costs for CD4 counts decreased due to fewer CD4 counts performed, resulting in a 68% reduction in annual costs: pre-EmERGE CD4 counts accounted for 31% of annual costs, which decreased to 14% in the post-EmERGE period.

### Primary and secondary outcomes pre- and post- mHealth

**Figure 1:**
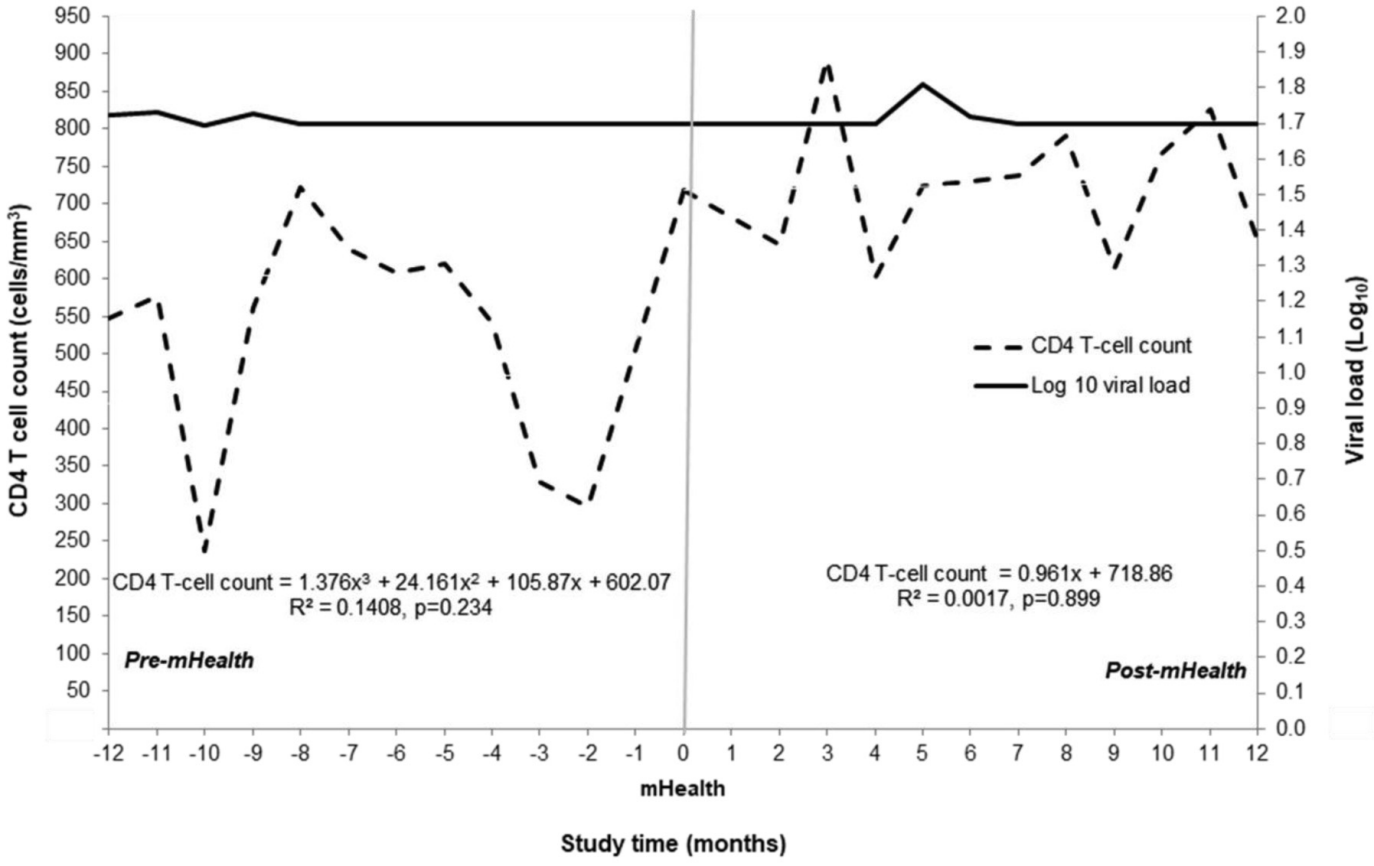
Mean CD4 cell count and HIV viral load at implementation of EmERGE Pathway (month 0) and twelve months before and after the start of follow-up (log10 1.7 < 50 copies/mL HIV-1 RNA).

The median CD4 cell count at study entry was 683 cells/mm^3^ (IQR 519-913 cells/mm^3^), and all but two participants had viral loads <50 copies/mL (log10 1.7); shortly after recruitment viral loads for these two participants became undetectable. Average CD4 count before EmERGE was 602 cells/mm^3^ compared with 719 cells/mm^3^ after EmERGE. Viral loads remained undetectable before and after EmERGE (Figure 1).

Measures of patient activation (PAM13) and quality of life (PROQOL-HIV) did not change substantially in the year after EmERGE implementation (Table 5).

**Table 5:** Median and interquartile range for PAM13 and PROQOL-HIV at months 0 and 12 post-mHealth implementation at University Hospital for Infectious Diseases

	Pre-EmERGE			Post-EmERGE		
	n	median	interquartile range	n	median	interquartile range
**PAM 13**		67.8	58.1 to 80.9		67.8	60.6 to 77.7
**PROQOL-HIV**						
**Physical health and symptoms**	302	88.9	75.0 to 97.2	246	87.5	75.0 to 97.2
**Body change**	302	93.8	75.0 to 100.0	249	93.8	75.0 to 100.0
**Social relationships**	306	100.0	87.5 to 100.0	249	100.0	87.5 to 100.0
**Intimate relationships**	304	75.0	58.3 to 100.0	247	83.3	58.3 to 100.0
**Stigma**	305	50.0	12.5 to 75.0	248	50.0	25.0 to 75.0
**Emotional distress**	305	87.5	68.8 to 100.0	250	87.5	75.0 to 100.0
**Health concerns**	304	75.0	56.3 to 93.8	250	75.0	56.3 to 93.8
**Treatment impact**	302	87.5	75.0 to 92.5	249	87.5	75.0 to 95.0

## Discussion

This analysis demonstrated the cost savings associated with the implementation of the EmERGE Pathway for stable PLHIV in the UHID HIV clinic. The 17% reduction across all outpatient visits and services resulted in a 33% cost reduction. Combined with the costs of ARVs, average annual cost decreased by 5%, from 43 101 HRK (95%CI 42 728-43,490) to 40 743 HRK (95%CI 40 466-41,034). Apart from demonstrating the cost-savings associated with the implementation of EmERGE, this is the first study producing contemporary annual cost for managing stable PLHIV in Croatia. Primary and secondary outcome measures did not change substantially during the study (31).

The reduction in annual costs was due to fewer outpatient visits and the performance of fewer tests. A malfunction of the CD4 flow-cytometer during the post-EmERGE period resulted in fewer CD4 counts performed and reduced costs. After the study, the clinic policy on performing CD4 counts changed. Routine CD4 counts for medically stable PLHIV with undetectable HIV-1 viral load are now routinely performed every two years depending on previous CD4 counts, adherence to anti-retroviral therapy (ART), presence of co-morbidities, and the psychological status of the PLHIV.

Apart from changing the routine measurements of CD4 counts in stable PLHIV, the introduction of the Pathway also changed the way staff interacted with participants, which may have also resulted in cost changes. This was unfortunately not quantified during the study, but medical staff indicated that they were able to spend more time on PLHIV with more complex disease (32).

The Pathway was well received by users. When asked, most participants preferred to use the App at home and away from their work (32). This reduced the risk of disclosure and empowered them. Some privacy concerns remained, especially among black and migrant women, but most agreed that the Pathway provided greater privacy. Some remained anxious that the App could be seen on the phone by friends or family members, who could then ask questions (32).

The App provided the participants with autonomy, as they depended less on visiting the clinic for routine consultations. Most participants found the test results function the most important; participants, including women and migrants, enjoyed having the EmERGE mHealth App and following the new Care Pathway (32).

The App reduced traveling and waiting times. While virtual sessions were more formal and more focused on results, they were also less likely to be interrupted. Face-to-face meetings, however, provided a better opportunity to develop close relationships, and facilitate open dialogue and negotiations over complex tasks (32).

ARVs were the major cost-drivers in the UHID HIV clinic as in the other four EmERGE clinics. Converting the costs into US dollars based on 2017 Gross Domestic Product (purchasing parity prices), an index of a country’s wealth (33), the annual cost of ARVs in this Hospital was US$ 10 671, compared with US$9595 to US$17 230 per year for the other EmERGE sites (34).

Outpatient costs could be further reduced by switching to quality-assured and affordable generic forms of the ARVs, although the use of generic drugs is not without its own issues (35). Using a single-daily-pill regimen also reduces health care costs (36), especially generic single-pill regimens.

During the costing exercise, UHID staff were very cooperative. Few data were available on the financial support, especially those provided by overhead departments, and assumptions had to be made to apportion costs (21); staff costs could only be broken down for the outpatient department. Assumptions had to be made to apportion costs between units of the Immunology and Molecular Diagnostics Department. Similarly, for the equipment costs of the Biochemistry and Hematology Laboratories and SCOPE cost data for the Radiology Department could not be obtained. The accounting system in the Hospital needs to be improved to allow access to more detailed financial information for future studies to assess and improve the efficiency of hospital services.

The status of the health information systems delayed collecting information on departmental workload. Outdated systems of storing data were used and there was limited sharing of data between departments. Different sources sometimes produced different information, especially data on departmental workload; conflicting data had to be reconciled. Data confidentiality was an issue that initially delayed data collection, but departmental heads were eventually convinced that their data would remain confidential and secure. At the end of the site visit, the need to integrate the various sections of the hospital’s health information system was started to be addressed. As PLHIV are living longer they are likely to develop co-morbidities and need follow-up in different hospital departments. Patient-level information should be linked across the site, while protecting the confidentiality and security of these data.

For mHealth to be successful, information at rest – on the person’s phone or the institutional server(s) – in-transit, and during its use, need to have their confidentiality and security protected (37). The range of potential issues and solutions for protecting personal health information have been described elsewhere (13). Paper-based and electronic tools have been developed and implemented to investigate the existence and implementation of national guidance, and adherence to them, to protect personal health information at facility, data warehouse, and national levels (38). Protocols to achieve such protection should be regularly reviewed, adapted, and improved.

Over the last decades, systematic reviews have analyzed mHealth interventions. Many highlight the variable effectiveness of mHealth tools (14). The EmERGE study was successfully implemented in five different European countries, which demonstrates its applicability in different cultural settings.

HIV-specific (8,9,39) and mHealth tools for other chronic diseases (16,40,41) have been studied. Most studies were performed in high income countries, though the use of mHealth is increasingly promoted in low- and middle-income countries (42-47). However, conclusions drawn more than two decades ago remain relevant today: “*Most of the studies analysed were small scale, short term, pragmatic evaluations that added little to our knowledge of the costs and benefits that would be expected to result from the introduction of telemedicine services into routine clinical practice”* (14). The EmERGE study involved more participants across different health care systems and countries, but only involved medically stable PLHIV and follow-up was still relatively short.

Future efficiencies can be anticipated, and resources saved once the EmERGE Pathway is extended to other PLHIV. If these saving are well harnessed, resources can be provided to address other needs. Extending the use of the EmERGE Pathways should be systematically and closely monitored and their implementation evaluated (22). Funding should be sought from national or international agencies to monitor and evaluate changes in service provision.

mHealth has been successfully used as part of programs tackling the COVID-19 pandemic (48-50). Funding should go toward development and implementation of similar pathways for other diseases or developing common pathways for people with chronic conditions that are supplemented by disease-specific information, as mHealth tools can be used to deal with acute or chronic health issues (10,11). All add to the broader developments of health information systems. Tracking the use, cost, outcome, and impact of health services across facilities and linking of personal health information over time is an important part of providing Universal Health Coverage (51).
